# Dietary Factors Associated with Depressive Symptoms in Midlife Women 40–50 Years of Age Living in the United States

**DOI:** 10.1089/whr.2024.0107

**Published:** 2024-12-06

**Authors:** Holly J. Jones, Natalie Ledesma, Alex Gomez, Rochelle Zak, Kathryn A. Lee

**Affiliations:** ^1^ Martha S. Pitzer Center for Women, Children, and Youth, College of Nursing, The Ohio State University, Columbus, Ohio, USA.; ^2^ Cancer Resource Center, UCSF Helen Diller Family Comprehensive Cancer Center, San Francisco, California, USA.; ^3^ Department of Medicine, San Francisco VA Healthcare System, San Francisco, California, USA.; ^4^ Sleep Disorders Center, University of California San Francisco, San Francisco, California, USA.; ^5^ Department of Family Health Care Nursing, School of Nursing, University of California, San Francisco, California, USA.

**Keywords:** vitamin C, fiber, supplements, saturated fat, omega-3, omega-6, sucrose, galactose, premenopause, sugar

## Abstract

**Purpose:**

Women in the decade before menopause are at risk for depression. This study describes dietary factors associated with depression risk in late premenopausal women that could be modifiable with targeted interventions.

**Methods:**

Descriptive cross-sectional study comparing a community-based sample of 342 healthy premenopausal women categorized as low-risk and high-risk for depression in a secondary analysis of dietary variables. Depression risk was estimated with the Center for Epidemiological Studies-Depression (CES-D) scale. Dietary variables were based on three random 24-hour diet recalls during a 1-week period that included an in-person visit with measures of potential covariates such as blood pressure, height, and weight for body mass index (BMI), a urine sample for follicle-stimulating hormone, demographic factors, exercise and sleep. Independent *t*-tests were used initially to compare groups, followed by logistic regression to adjust for covariates. Statistical significance was set at *p* ≤ 0.05.

**Results:**

Depression risk (CES-D ≥ 16) was present in 30% of participants. Compared with the low-risk group, the high-risk group had significantly higher intake of polyunsaturated fat, omega-6 linoleic acid and sucrose, and lower intake of galactose, vitamin C, and omega-3 eicosapentaenoic acid. After adjusting for energy intake and significant covariates (income adequacy, education, race/ethnicity, marital status, BMI, exercise and sleep duration), only polyunsaturated fat, omega-6, and sucrose remained significant.

**Conclusion:**

Depression prior to menopause is common and multifactorial. Findings support the importance of assessing saturated fats, omega-6 fatty acids, and sucrose. Attention to diet in addition to exercise and sleep may improve intervention outcomes for mental health in midlife women.

## Introduction

Depressive symptoms are experienced by 18%–19% of adults in the United States.^1^ Vulnerability increases for adults over 40 years old and is consistently higher for women than men of the same age.^2,3^ Depressive symptoms for women during midlife, 40–60 years of age, have been explored in relation to hormonal changes and family issues such as limited financial and social resources, divorce, widowhood, or children leaving home.^4,5^ Evidence also suggests that depression is associated with modifiable health factors such as lack of physical activity, inadequate sleep, and obesity.^5^ Western dietary patterns include consumption of ultra-processed foods associated with depressive symptoms in the general population^6^,**^7^** with notable sex differences in fiber for women and sugar for men^8^ and lower mineral intake for women but not for men.^9^ The relationship between postmenopausal depression and intake of sugar and fiber has also been noted in U.S. studies such as the Women’s Health Initiative^10^ and, in women 42-52 years old in the Nurses’ Health Study.^11^

Very little research on dietary factors and depressive symptoms has been conducted in premenopausal women. In the Study of Women Across the Nation (SWAN), results differed by menopausal status for some vitamins^12^ and vitamin C was associated with depressive symptoms when menopause status was controlled.^13^ In a large Australian sample of young women, 4%–6% were categorized as depressed, and depression was associated with general diet quality^14^ but not with specific fruit or vegetable intake.^15^ However, little research has been done to describe dietary factors before menopause onset when additional factors begin to complicate the experience of depressive symptoms. The purpose of this study was to identify dietary factors associated with depression risk in late premenopausal women aged 40–50 years. Based on prior literature, and controlling for risk factors such as education, income, race/ethnicity, partner status, smoking, exercise, sleep, and body mass index (BMI), we hypothesized that women with higher risk for depression would have lower intake of vitamins and minerals as well as higher intake of fat, carbohydrates, and sugar compared with women at low risk for depression.

## Methods

### Participants

Healthy women aged 40–50 years living in the San Francisco Bay Area participated in the University of California San Francisco Midlife Women’s Health Study. We enrolled women self-identifying as White, Black, or Latina, still experiencing regular menstrual cycles and not planning a pregnancy. We excluded women taking hormone therapy or reporting a major health problem such as cancer, stroke, diabetes, sleep disorder, depression diagnosis, or recent hospitalization. Details about recruitment and physical activity were previously reported.^16^ The institution’s Committee on Human Research approved the study and participants provided informed consent. Of the 347 participants, 5 were excluded for pregnancy, surgical menopause, missing depression scores, and an invalid diet recall.

### Measures

#### Depressive symptoms

We assessed frequency of depressive symptoms with the 20-item Center for Epidemiological Studies–Depression (CES-D) scale, used to screen for depression in the general population^17^ with adequate validity and reliability in middle-aged adults.^18^ Participants are asked how often they experienced symptoms, for example “*I had crying spells*” or “*I felt sad*” during the past week on a scale of 0 (*rarely/none or 1 day*) to 3 (*most/all the time or 5–7 days*). The CES-D total score ranges from 0 to 60 and ≥16 suggests risk for depression and need for clinical evaluation.^17^ Cronbach alpha internal consistency reliability for the 20 items was 0.91 in this sample.

#### Dietary variables

Participants completed the National Cholesterol Educational Program (NCEP) questionnaire representing eight food categories: meat, eggs, dairy, fried foods, baked goods, convenience foods, table fats, and snacks (MEDFICTS).^19^ We showed food models to participants and asked them to indicate consumption and serving sizes for the past week. The NCEP identifies diets low in saturated fat (Step II diet, <7% calories from saturated fat, <40 points). Scores ranged from 0 (no saturated fat) to 132 (high saturated fat). Participants also completed an investigator-developed checklist for supplements, special diet or dietary restrictions, plans to diet and reasons for any recent weight gain or loss.

We obtained dietary intake of nutrients from three 24-hour recalls supplemented with questions about items from a Food Frequency Questionnaire such as milk in cereal or coffee, mayonnaise, and butter or margarine on vegetables, potatoes, noodles, or rice. For the first diet recall, food models were visibly displayed at the home visit; second and third diet recalls were obtained at random during the following week by telephone. In addition to calories from protein, fat, and carbohydrates, we analyzed nutrients from food, excluding vitamin or mineral supplements using the Nutritionist-4 software (First Databank Inc., Hearst Corp, San Bruno CA), with over 70 nutrients from a wide variety of ethnic foods and fast foods in its database. We considered any diet recall <800 kcal/day invalid and excluded it from calculations of nutrient intake. In 1041 recalls, 71 (6.8%) were invalid; one participant was excluded because all three recalls were invalid.

#### Sociodemographic and health risk factors

Race/ethnicity was self-identified at telephone screening. The demographic questionnaire at the home visit included questions about education, employment, smoking, marital status, mean household income, and adequacy of income for food and other essentials such as housing and health care. Responses ranged from 1 (“*not at all adequate*”) to 4 (“*more than adequate*”).

At the home visit, we obtained a first-morning urine sample for follicle-stimulating hormone (FSH) to assess menopausal status and obtained weight with a battery-operated calibrated scale, accurate to 0.1 pound (Precision Health Scale Model UC-300). We obtained height with a portable stadiometer (Accustat, Scale-Tronix, Inc., Carol Stream, IL) without shoes. BMI was computed as weight (kg) divided by height (m) squared (kg/m^2^).

#### Physical activity and sleep duration

Physical activity was estimated using the Paffenbarger Physical Activity Questionnaire (PPAQ), a self-report measure about activities during the past week. The PPAQ question about days in the past week the participant exercised to work up a sweat was used as a covariate.^20^ Results for physical activity were previously reported.^16^

Our final covariate was sleep duration assessed with a self-report item from the Pittsburgh Sleep Quality Index (PSQI) that asks about the typical hours of sleep per night during the past month.^21^ This PSQI item was correlated with the PPAQ item for resting/reclining on weekdays (*r* = 0.611, *p* < 0.001) and weekends (*r* = 0.380, *p* < 0.001).

### Statistical analysis

Descriptive statistics included means with standard deviations (SD), and frequency distributions with percentages. We categorized each participant as low-risk for depression (CES-D score <16) or high-risk for depression (CES-D ≥ 16) and tested for group differences with *t*-tests for continuous variables and chi square for categorical variables. To describe depression risks associated with poor nutrition, we used logistic regression with CES-D group as the dependent outcome and adjusted for caloric intake, demographic factors (race/ethnicity, partner status, education, income adequacy for food), and health factors (BMI, exercise, sleep duration). We performed all analyses using SPSS version 22.0. Statistical significance was set at *p* ≤ 0.05.

## Results

The overall mean age was 43.4 ± 3.4 years. Participants had regular cycles and FSH levels consistent with premenopausal stage (<2.5 IU/dL). Most (86%) were employed, 58% had a college degree, and only 10% smoked cigarettes. The mean CES-D score was 12.4 ± 9.76 (median = 10) and we categorized 104 (30%) as high-risk for depression (≥16). In unadjusted comparisons (Table 1), the high-risk group had significantly higher BMI, less frequent exercise, shorter sleep duration, and less adequate income for food. The high-risk group was also significantly more likely to be White and less likely to be partnered or complete college. In adjusted logistic regression analysis, (Table 1) the CES-D risk group remained associated with education, exercise, and sleep duration, whereas BMI was no longer significant. Age, employment status, hypertension, smoking, use of vitamin or mineral supplements, and FSH were not significant.

**Table 1.** Sociodemographic and Health Characteristics by Depressive Symptom Risk Group

	Depressive symptom group			
	Low-risk CES-*D* < 16 (*n* = 238; 70%)	High-risk CES-D ≥ 16 (*n* = 104; 30%)	Unadjusted comparisons	Adjusted^b^ logistic regression
Characteristic	Mean ± SD or *n* (%)	Mean ± SD or *n* (%)	Statistic, *p* value	*Wald* statistic, *p* value
CES-D score	7.2 ± 4.6	24.5 ± 7.6	—	—
Age (years)	43.4 ± 2.4	43.5 ± 2.3	*t(340*) = 0.028, *p* = 0.978	*Wald* = 0.008, *p* = 0.927
FSH (IU/dl)	0.96 ± 1.90	1.26 ± 2.27	*t(340)* = −1.239, *p* = 0.216	*Wald* = 0.825, *p* = 0.364
Hypertension (≥140 mmHg systolic or ≥89 mmHg diastolic)	13 (5.5%)	10 (9.6%)	χ^2^ (1) = 1.96, *p* = 0.161	*Wald* = 0.781, *p* = 0.377
Take vitamin supplements	131 (55%)	62 (60%)	χ^2^(1) = 0.62, *p* = 0.433	*Wald* = 0.981, *p* = 0.322
Take mineral supplements	108 (45%)	46 (44%)	χ^2^(1) = 0.04, *p* = 0.844	*Wald =* 0.534, p = 0.465
Smoke cigarettes	23 (10%)	13 (13%)	χ^2^(1) = 0.62, *p* = 0.432	*Wald =* 0.120, p = 0.729
Race/ethnicity			χ^2^(2) = 10.05, *p =* **0**.**007**	*Wald* = 1.913, *p* = 0.167
African American	49 (20%)	38 (37%)		
Latina	71 (30%)	22 (21%)		
White	118 (50%)	44 (42%)		
College graduate	150 (63%)	47 (46%)	χ^2^(1) = 7.92, *p =* **0**.**005**	*Wald =* 3.916, **p = 0.048**
Partnered or married	146 (61%)	50 (49%)	χ^2^(1) = 4.44, *p =* **0**.**035**	*Wald* = 0.246, *p* = 0.620
Employed	202 (86%)	83 (86%)	χ^2^(1) = 0.01, *p =* 0.926	*Wald* = 0.633 *p =* 0.426
Income adequacy for food (1–4)	3.24 ± 0.62	3.06 ± 0.69	*t(340*) = 2.434, *p* = **0**.**015**	*Wald* = 2.090, *p* = 0.148
Body mass index (kg/m^2^)	26.7 ± 5.7	28.7 ± 7.5	^^a^^*t*(156.1) = −2.435, *p* = **0**.**016**	*Wald =* 0.614, p = 0.433
Exercise (days/week)	2.20 ± 1.98	1.57 ± 1.97	*t(340*) = 2.735, *p* < 0.007	*Wald =* 5.913, **p = 0.015**
Sleep duration (h)	6.99 ± 0.88	6.23 ± 1.12	^^a^^*t*(173.6) = 5.586, *p* < 0.001	*Wald* = 23.44, *p* < 0.001

Bolded *p* values indicate significant association (*p ≤* 0.05).

^a^ Separate variance *t*-test with adjusted degrees of freedom due to unequal variances.

^b^ Logistic regression model adjusted for 15 variables: age, FSH, White race yes/no, education, income adequacy for food, partner, employed, exercise days, BMI, sleep duration, smoking, hypertension, vitamin supplements, mineral supplements, and calorie intake.

CES-D, Center for Epidemiological Studies-Depression Scale; FSH, urinary sample for follicle-stimulating hormone; SD, standard deviation.

### Diet characteristics

Average caloric intake based on three diet recalls was 1600 kcal/day. Both groups were similar on kcals from protein (17%), carbohydrates (52%–53%), and fat (30%–31%), and 51% (*n* = 174) followed a healthy Step II diet with NCEP scores reflecting <7% saturated fat. For purposes of validity, NCEP scores were correlated with diet recall results for fat intake (*rho* = 0.336, *p* < .001) and cholesterol (*rho* = 0.240, *p* < 0.001). There were no group differences for total calories, fiber, net carbohydrates, or caffeine (Table 2). Intake of alcohol was skewed, with most reporting no alcohol. The high-risk depression group consumed significantly less alcohol, but that difference disappeared when adjusted for covariates (Table 2).

**Table 2.** Dietary Characteristics by Center for Epidemiological Studies-Depression Scale Depressive Symptom Risk Group

	Depressive symptom group			
	Low-risk (*n* = 238)	High-risk (*n* = 104)	Unadjusted comparisons	Adjusted^b^
Dietary characteristic	Mean ± SD	Mean ± SD	*t* statistic, *p* value	*Wald* statistic, *p* value
Kilocalories	1590 ± 388	1676 ± 475	^^a^^*t*(165.6) = −1.628, *p* = 0.105	^c^*Wald =*1.458, *p =* 0.227
Protein (g)	64.7 ± 19.3	67.6 ± 26.5	^^a^^*t*(152.6) = −1.000, *p* = 0.319	*Wald =* 0.193, p = 0.661
Carbohydrates (g)	208 ± 62	217 ± 64	*t(340)* = −1.137, *p* = 0.256	*Wald =* 0.088, p = 0.766
Fiber (g)	15.6 ± 6.5	15.0 ± 8.2	^^a^^*t*(162.8) = 0.623, *p* = 0.534	*Wald* = 0.032, *p =* 0.858
Net carbohydrates (g)	192 ± 59.4	203 ± 59.5	*t(340)* = −1.281, *p* = 0.201	*Wald =* 0.078, p = 0.781
NCEP dietary fat score	39.3 ± 23.0	48.2 ± 26.8	*t(340)* = −2.751, *p* = **0**.**006**	*Wald =* 0.501, p = 0.479
Fat (g)	53.1 ± 19.8	60.3 ± 27.3	^^a^^*t*(152.4) = −2.422, *p* = **0**.**017**	*Wald =* 1.926, p = 0.165
Saturated fat (g)	16.9 ± 7.9	18.9 ± 10.4	^^a^^*t*(156.5) = −1.726, *p* = **0**.**086**	*Wald =* 0.117, p = 0*.674*
Monounsaturated fat (g)	16.6 ± 7.4	18.6 ± 9.3	^^a^^*t*(161.7) = −1.955, *p* = **0**.**052**	*Wald =* 1.154, p = 0.283
Polyunsaturated fat (g)	9.9 ± 4.7	11.5 ± 6.8	^^a^^*t*(152.4) = −2.142, *p* = **0**.**034**	*Wald =* 4.867, p = **0.027**
Cholesterol (mg)	210 ± 129	225 ± 130	*t(340)* = −0.983, *p* = 0.326	*Wald =* 0.008, p = 0.928
ALA omega-3 (g)	0.618 ± 0.400	0.554 ± 0.372	*t(340*) = 1.389, *p* = 0.166	*Wald =* 1.490, p = 0.222
EPA omega-3 (mg)	36.2 ± 96.2	15.7 ± 32.4	^^a^^*t*(325.4) = 2.934, *p* = **0**.**004**	*Wald =* 3.516, p = **0.061**
DHA omega-3 (mg)	78.8 ± 147.8	54.8 ± 82.5	^^a^^*t*(320.6) = 1.913, *p* = **0**.**057**	*Wald =* 1.460, p = 0.227
Omega-6 Linoleic acid (g)	6.25 ± 3.72	7.50 ± 5.07	^^a^^*t*(153.4) = −2.256, *p* = **0**.**025**	*Wald =* 5.783, p = **0**.**016**
Omega-9 Oleic acid (g)	12.0 ± 5.7	12.5 ± 6.6	*t(340)* = −0.577, *p* = 0.656	*Wald =* 0.301, p = 0.583
Caffeine (mg)	155 ± 166	156 ± 161	*t(340*) = 0.087, *p* = 0.930	*Wald =* 0.221, p = 0.638
Alcohol (g)	5.59 ± 9.21	3.56 ± 7.00	^^a^^*t*(255.1) = 2.219, *p* = **0**.**027**	*Wald =* 1.378, p = 0.240
Total sugars (g)	78.0 ± 38.5	84.8 ± 37.1	*t(340)* = −1.504, *p* = 0.134	*Wald* = 0.032, *p* = 0.859
Fructose (g)	13.5 ± 9.6	14.0 ± 9.1	*t(340)* = −0.481, *p* = 0.631	*Wald =* 0.002, p = 0.962
Galactose (g)	0.27 ± 0.53	0.14 ± 0.32	^^a^^*t*(310.1) = 2.755, p = **0**.**006**	*Wald =* 2.829, p = 0.093
Glucose (g)	12.6 ± 9.1	12.8 ± 8.1	*t(340)* = −0.189, *p* = 0.851	*Wald =* 0.006, p = 0.937
Lactose (g)	6.12 ± 5.69	7.00 ± 9.22	^^a^^*t*(138.5) = −0.934, *p* = 0.352	*Wald =* 2.001, p = 0.157
Maltose (g)	1.43 ± 2.02	1.47 ± 2.03	*t(340)* = −0.154, *p* = 0.878	*Wald =* 0.242, p = 0.623
Sucrose (g)	16.3 ± 12.1	21.0 ± 19.0	^^a^^*t*(140.8) = −2.336, *p* = **0**.**021**	*Wald =* 6.216, p = **0.013**

^a^ Separate variance *t*-test with adjusted degrees of freedom due to unequal variance.

^b^ Logistic regression adjusted for caloric intake, race, education, income for food, partner, BMI, exercise, and sleep duration.

^c^ Calorie intake adjusted for age, FSH, race, education, income for food, partner, employed, exercise, BMI, sleep duration, smoking, hypertension, vitamin supplements, and mineral supplements.

ALA, α-linolenic acid; BMI, body mass index; CES-D, Center for Epidemiological Studies-Depression Scale; DHA, docosahexaenoic acid; EPA, eicosapentaenoic acid; FSH, follicle-stimulating hormone; NCEP, National Cholesterol Education Program.

### Group differences in fat intake

In unadjusted group comparisons (Table 2), the high-risk depression group had a significantly higher NCEP score (*p* = 0.006), indicating a diet high in saturated fat. The high-risk group also had significantly higher intake of fat, polyunsaturated fat, and the omega-6 fatty acid, linoleic acid. Intake of each omega-3 fatty acid was lower in the high-risk group, but only EPA was significantly lower, while DHA had a trend (*p* = 0.057) and ALA was not significantly less (*p* = 0.166). There was no group difference in cholesterol intake.

For adjusted analysis we controlled for kcals and the seven salient sociodemographic and health factors identified as significant in Table 1 (BMI, exercise, income for food, race/ethnicity, education, sleep, and partner). After adjusting for all eight covariates, higher intake of total polyunsaturated fat and linoleic acid remained significant (*p* = 0.016), there was a trend for less EPA omega-3 fatty acid (*p* = 0.061) and overall fat intake and saturated fat diet scores were no longer significant (Table 2).

### Group differences in sugar intake

In addition to overall sugar intake, we compared six specific sugars (Table 2). Overall sugar intake did not differ by group, but the high-risk group consumed significantly less galactose and more sucrose (Table 2). After adjusting for the eight covariates, intake of sucrose remained significant (*p* = 0.013) and galactose was no longer significant (*p* = 0.093).

### Groups differences in vitamins, minerals, and amino acids

In unadjusted and adjusted comparisons we found no vitamin or mineral that significantly distinguished the high-risk group from the low-risk group (Table 3). There were also no group differences in intake of essential and non-essential amino acids (Table 4). Because of the trend for a group difference in vitamin C intake before adjusting for covariates (*p* = 0.089), we explored median group differences: the low-risk group had significantly (Mann–Whitney *U, p* = 0.026) higher median intake of vitamin C (97 mg) than the high-risk group (77 mg).

**Table 3.** Intake of Vitamins and Minerals by Center for Epidemiological Studies-Depression Scale Depressive Symptom Risk Group

	Depressive symptom group			
	Low-risk (*n* = 238)	High-risk (*n* = 104)	Unadjusted comparisons	Adjusted^b^
Vitamins	Mean ± SD	Mean ± SD	*t* statistic, *p* value	*Wald* statistic, *p* value
Beta-carotene (µg)	526 ± 792	467 ± 687	*t(340*) = 0.660, *p* = 0.509	*Wald =* 0.644, p = 0.422
Vitamin A (RE)	959 ± 827	965 ± 839	*t(340)* = −0.057, *p* = 0.955	*Wald* = 0.082, *p* = 0.775
Vitamin C (mg)	107.8 ± 69.0	93.9 ± 70.7	*t(340*) = 1.706, *p* = **0**.**089**	*Wald* = 1.815, *p* = 0.178
Vitamin D (µg)	2.01 ± 2.07	2.07 ± 2.05	*t(340)* = −0.248, *p* = 0.804	*Wald =* 0.526, p = 0.468
Vitamin E (mg)	5.94 ± 4.13	6.97 ± 6.05	^^a^^*t*(146.6) = −1.575, *p* = 0.117	*Wald =* 1.318, p = 0.251
α-Tocopherol (mg)	1.28 ± 2.13	1.09 ± 2.00	*t(340*) = 0.757, *p* = 0.449	*Wald =* 0.007, p = 0.935
Vitamin K (µg)	90.9 ± 74.5	89.8 ± 104.8	*t(340*) = 0.112, *p* = 0.911	*Wald =0.490, p =* 0*.484*
B1Thiamin (mg)	1.26 ± 0.54	1.20 ± 0.57	*t(340*) = 1.003, *p* = 0.317	*Wald =1.901, p =* 0*.168*
B2 Riboflavin (mg)	1.32 ± 0.49	1.28 ± 0.64	^^a^^*t*(157.0) = 0.542, *p* = 0.589	*Wald =0.620, p =* 0*.431*
B3Niacin (mg)	17.5 ± 6.57	17.7 ± 7.52	*t(340)* = −0.258, *p* = 0.797	*Wald =0.225, p =* 0*.635*
B6 Pyridoxine (mg)	1.37 ± 1.59	1.30 ± 0.65	*t(340*) = 1.067, *p* = 0.287	*Wald =0.912, p =* 0*.340*
B9 Folate (µg)	222.2 ± 110.8	215.0 ± 133.0	*t(340)* =-0.518, *p* = 0.605	*Wald = 0.011, p =* 0*.916*
B12 Cobalamin (µg)	2.82 ± 2.04	2.85 ± 2.40	*t(340)* = −0.128, *p* = 0.898	*Wald =0.162, p =* 0*.687*
Minerals				
Calcium (mg)	652 ± 288	632 ± 342	*t*(*340*) = 0.563, *p* = 0.574	*Wald* = 0.331, *p* = 0.565
Copper (mg)	0.96 ± 0.38	0.93 ± 0.46	^^a^^*t*(167.0) = 0.594, *p* = 0.554	*Wald =* 0.114, p = 0.735
Iron (mg)	12.4 ± 5.4	12.1 ± 5.5	*t*(*340*) = 0.517, *p* = 0.606	*Wald =* 1.008, p = 0.315
Magnesium (mg)	223 ± 75.4	228 ± 112.9	^^a^^*t*(144.7) = −0.357, *p* = 0.722	*Wald =* 0.711, p = 0.399
Manganese (mg)	2.25 ± 1.04	2.21 ± 1.43	*t(340*) = 0.288, *p* = 0.773	*Wald =* 0.233, p = 0.630
Phosphorus (mg)	909.3 ± 292.2	895.3 ± 396.2	*t*(*340*) = 0.366, *p* = 0.714	*Wald =* 0.646, p = 0.422
Potassium (mg)	2248 ± 728	2156 ± 888	*t(340*) = 1.005, *p* = 0.315	*Wald* = 0.543, *p* = 0.461
Selenium (mg)	0.046 ± 0.026	0.048 ± 0.027	*t(340*) = 0.651, *p* = 0.516	*Wald =* 0.375, p = 0.541
Sodium (mg)	2273 ± 856	2322 ± 1080	*t(340)* = −1.364, *p* = 0.173	*Wald* = 0.036, *p* = 0.850
Zinc (mg)	7.51 ± 3.61	7.07 ± 3.47	*t(340*) = 1.043, *p* = 0.298	*Wald =* 1.533, p = 0.216

^a^ Separate variance *t*-test with adjusted degrees of freedom due to unequal variance.

^b^ Logistic regression adjusted for caloric intake, race, education, income for food, partner, BMI, exercise, and sleep duration.

**Table 4.** Essential and Nonessential Amino Acid Comparisons by Depressive Symptom Risk Group

	Depressive Symptom Group		
	Low-risk (*n* = 238)	High-risk (*n* = 104)	Group comparisons
Essential amino acids	Mean ± SD	Mean ± SD	t statistic, *p* value
Histidine (mg)	1369 ± 591	1392 ± 690	*t(340)* = −0.315, *p* = 0.753
Isoleucine (mg)	2311 ± 940	2386 ± 1233	*t*(*340)* = −0.609, *p* = 0.543
Leucine (mg)	3813 ± 1508	3906 ± 1981	*t*(*340)* = −0.474, *p* = 0.636
Lysine (mg)	3353 ± 1545	3425 ± 1850	*t*(*340)* = −0.370, *p* = 0.712
Methionine (mg)	1133 ± 490	1170 ± 626	*t*(*340)* = −0.603, *p* = 0.547
Phenylalanine (mg)	2145 ± 811	2221 ± 1130	*t*(*340)* = −0.698, *p* = 0.486
Threonine (mg)	1926 ± 815	1981 ± 1010	*t*(*340)* = −0.534, *p* = 0.594
Tryptophan (mg)	576 ± 231	594 ± 312	*t*(*340)* = −0.576, *p* = 0.565
Valine (mg)	2536 ± 987	2618 ± 1375	^^a^^*t*(_151.3)_ = −0.622, *p* = 0.534
Nonessential amino acids			
Alanine (mg)	2079 ± 941	2104 ± 1073	*t*(*340)* = −0.217, *p* = 0.828
Arginine (mg)	2362 ± 1064	2353 ± 1208	*t*(*340)* = −0.701, *p* = 0.484
Aspartic acid (mg)	3681 ± 1593	3760 ± 1872	*t*(*340)* = −0.403, *p* = 0.687
Cysteine (mg)	665 ± 261	687 ± 350	*t*(*340)* = −0.624, *p* = 0.533
Glutamic acid (mg)	8170 ± 2907	8232 ± 3718	^^a^^*t*(160.4) = −0.153, *p* = 0.866
Glycine (mg)	1827 ± 878	1877 ± 935	*t(340)* = −0.475, *p* = 0.635
Proline (mg)	2747 ± 965	2750 ± 1321	^^a^^*t*(152.9) = −0.022, *p* = 0.982
Serine (mg)	1938 ± 727	1978 ± 984	^^a^^*t*(154.0) = −0.368, *p* = 0.714
Tyrosine (mg)	1697 ± 672	1742 ± 920	^^a^^*t*(153.0) = −0.451, *p* = 0.652

^a^ Separate variance *t*-test with adjusted degrees of freedom due to unequal variance.

## Discussion

For this secondary analysis, we hypothesized that high risk of depression would be associated with dietary factors after controlling for known risk factors such as obesity, food insecurity, exercise, sleep duration, and FSH level as an indicator of reproductive stage. Our overall sample had CES-D scores similar to the SWAN midlife sample^5^ and 30% were high-risk for depression. Our unadjusted results indicated significant group differences in race/ethnicity, education, marital status, food insecurity, BMI, exercise, and sleep duration; only education, exercise, and sleep remained significant in logistic regression models. While it was surprising that smoking, employment, age, and FSH did not differ by depression risk group, few participants smoked, most were employed, and all were premenopausal within a narrow age range. Income adequacy for food, our measure of food insecurity, was no longer significant in multivariate analysis. In contrast with prior studies that typically included large samples of adults from wide geographic areas^6^ our participants were from one geographic location with a mild climate and similar access to fresh foods. BMI and race were no longer significant in multivariate analysis, indicating that other modifiable health factors, such as exercise and sleep, may have more influence on depressive symptoms than obesity, race/ethnicity, or food insecurity. As detailed below, we found higher risk of depression to be significantly associated with higher intake of polyunsaturated fats, omega-6 linoleic acid, and sucrose but not associated with vitamin or mineral intake.

### Saturated and unsaturated fats

Before adjusting for demographic and modifiable health factors, there were significant group differences in some dietary factors associated with ultra-processed foods, including more saturated fat and omega-6 linoleic acid but lessomega-3 fatty acids, especially EPA in the high-risk group. However, after adjusting for covariates, only polyunsaturated fat and omega-6 linoleic acid remained significant, with a trend in less EPA (*p* = 0.061). Others^22,23^ have shown depressive symptoms inversely associated with omega-3 fatty acids. We also found an association between depressive symptoms and omega-6 linoleic acid while others reported either an inverse relationship^23^ or an association with the ratio of omega-6:omega-3.^24^ These fatty acids may modulate depression through anti-inflammatory processes, as shown in the mouse model for depression with omega-3 reducing hippocampal inflammation.^25^

These data emphasize that attention to omega-6 and omega-3 fatty acids may be helpful in modulating depressive symptoms in mid-life women. Our small sample may have limited power for detecting statistical significance for omega-3 fatty acids, yet linoleic acid, an omega-6 fatty acid commonly found in plant oils used in processed food products, had clinically small group differences that remained statistically strong. In future studies, the ratio of omega-6 to omega-3 fatty acids should be examined more closely, as our high-risk group had a higher intake of one form of omega-6 and lower intake of three forms of omega-3 compared with the low-risk group.^26–28^

We analyzed food sources of only one form of omega-6 and three forms of omega-3 fatty acids and did not include nutritional supplements, which have mixed evidence for improving depressive symptoms.^29,30^ Because omega-6 fatty acids are involved in inflammation and immune function, more research is needed on depressive symptoms associated with inflammation and diets high in processed foods.^31^ Future research on fatty acids is warranted, particularly when considering the role of fatty acids in displacing tryptophan from albumin and tryptophan’s role in the serotonergic pathway of depression.^24,32^ Although we found no group difference for intake of protein or any amino acids, tryptophan’s potential role in sleep regulation is intriguing given that sleep duration was a consistently significant covariate in our multivariate analyses. Some discrepancy in the literature on diet and depression may be because few studies considered sleep duration a covariate.^31^

We found significantly higher fat intake, particularly saturated fats, in our high-risk group, but only polyunsaturated fat intake remained significant after adjusting for calorie intake and the other covariates. For a healthy diet, the Office of Dietary Supplements^33^ recommends <20 g saturated fat for women, or <10% of calories from saturated fat; our participants had a lower intake of saturated fat than seen in the general population, and dietary intake of cholesterol was also lower than the recommended <300 mg/day. This may reflect geographic location and consumer knowledge about healthy diets, yet supports ongoing consideration of education in nutrition research and the need for more educational programs to improve nutrition and mental health. High fat intake may indicate a diet high in processed foods, and while the literature is inconclusive on processed foods and depressive symptoms,^34–39^ a deeper understanding of dietary factors that might increase depressive symptoms is needed for women at risk for depression.^29^ In addition, controlling for health covariates such as sleep and exercise are critical for understanding depressive symptoms in midlife women.^40^

### Sugars

Similar to other studies on sugar and ultra-processed foods,^8,11^ we found that higher sucrose intake distinguished the high-risk group from the low-risk group, even after adjusting for demographic and modifiable health factors. Our finding is supported by longitudinal date in postmenopausal women whereby the highest intake of added sugar at baseline was significant for incidence of depression 3 years later.^10^ In a sample of adults over 50 years old, Chrzastek and colleagues^8^ found a relationship between depressive symptoms and sucrose intake for men (39.5 g) but not women (33.6 g). We also found a significant relationship with sucrose even though our sample of women consumed much less sucrose (<21 g). Because intake of sucrose reflects ultra-processed foods, our findings also support the Nurses’ Health Study findings for midlife women who completed a food frequency questionnaire every 4 years; a diet high in ultra-processed foods was a risk factor for depression.^11^

Of the three monosaccharides (glucose, fructose and galactose) analyzed, our high-risk group had significantly lower intake of galactose, a sugar associated with dairy products; this difference was no longer significant after adjusting for covariates. Gangwisch and colleagues^10^ did not report on galactose, but depression in postmenopausal women was associated with low lactose (disaccharide) intake in the Women’s Health Initiative sample. We found no group difference in lactose intake, although the high-risk group consumed, on average, 1 g more lactose.

Our findings of higher sucrose in the high-risk group would support testing behavioral interventions to reduce sugar intake in women experiencing depressive symptoms. Limiting the intake of processed foods, which are typically high in sucrose and fat, may be effective in lowering brain-derived neurotrophic factor that plays a key role in the limbic system’s regulation of mood and depressive symptoms.^24,31^

### Other macronutrients

We found no group differences for intake of fiber, carbohydrates, or protein. Our findings differ somewhat from prior studies^8,10^ and support others.^40^ Ebrahimpour-Koujan and colleagues^40^ found no relationship between depression and low-carbohydrate diets in a large sample of Iranian adults even after adjusting for sex, age, exercise, and BMI. They did not analyze net carbohydrate intake, but fiber differed significantly between their low-risk and high-risk mental health groups.^40^ Our finding of no difference in fiber intake differs from the Women’s Health Initiative sample where the lowest quintile differed from the highest quintile,^10^ but they also found no difference in overall carbohydrate intake. Our lack of difference in fiber also differs from Chrzastek and colleagues,^8^ who found sex differences; for women, depression was associated with low fiber intake. Our participants consumed fiber in amounts (15 g/day) similar to Chrzastek and colleagues’ sample (17 g/day) but lower than the recommended 25 g/day for women. More research is needed on depressive symptoms and the role of fiber and carbohydrates, particularly net carbohydrates rather than general intake of all carbohydrates.

### Vitamins

Based on prior studies of depressive symptoms in midlife women, we expected to find differences in vitamins^12,13^ but intake of B vitamins and vitamin supplements were similar in both group. Our negative findings contrast with a recent study in Brazil comparing women at lowest and highest quartiles^41^ and a meta-analysis specific to folate intake and serum levels,^42^ but our findings support a study comparing fruit and vegetable consumption in younger Australian women,^18^ a study of serum levels of folate and vitamin B12,^43^ and a meta-analysis on vitamin B12 supplementation.^44^ While our participants had the recommended intake of vitamin B12, mean intake of folate in both groups was only half the recommended 400 µg.^33^

Intake of vitamin C was higher in our sample than the recommended 75 mg/day^33^ and the trend for a group difference disappeared in adjusted analysis. These results conflict with the SWAN analysis of a diet recall without addressing geographical location or disparities in access to fresh fruit and vegetables.^13^ The SWAN sample was large and designed to represent diversity of women from seven different regions in the United States. However, diversity and food choices could be confounded by recruiting women of Japanese, Chinese, and Hispanic heritage at three separate sites, and recruiting African Americans at four of the seven sites.^4,5^ Medians for vitamin C intake in our sample (97 mg for low-risk and 77 mg for high-risk) were lower than medians for the SWAN sample (102 mg and 92 mg, respectively), and the difference between their extreme low and high quartiles was significant after adjusting for menopausal status, BMI, and other factors, but sleep duration and exercise were not included as covariates.^13^

Vitamin D intake from food was low in our sample compared with the 15 µg/day recommended for women by the Office of Dietary Supplements,^33^ and we found no group difference. Most literature on vitamin D focuses on supplements to prevent or treat depression with either negative findings^45^ or positive findings,^46,47^ and debate about cause and effect for depression must consider sun exposure, exercising indoors or outdoors, and use of sunscreen products.^48^

When considering conflicting results for vitamin intake, a confounding factor may include sex differences and reproductive status, which was controlled in our sample selection and analysis. Vitamin A intake differed for premenopausal and perimenopausal women in the SWAN sample,^12^ indicating that reproductive stage should be considered in nutrition and depression research. Furthermore, the Brazilian study indicated an association between depression and antioxidant vitamins A and C for women but not men.^41^ Based on a recent meta-analysis, the depression literature remains conflicting on antioxidants such as vitamin C and vitamin E even when sex differences are considered.^49^ Our negative findings for vitamins may be due to our sample’s mean intake above the amount recommended for vitamin C but below recommended amounts for vitamin A and vitamin E. Despite these conflicting findings, vitamins with their antioxidant properties need further research. From a public health perspective, there may be less resistance to depression interventions that use supplemental therapeutic doses of vitamin C for example, particularly when behavioral interventions such as weight loss or change in dietary habits are more difficult to adopt due to societal, personal, or cultural stigma.

### Minerals

Based on prior studies of depressive symptoms and mineral intake in women,^9^ we were surprised to find no differences in unadjusted or adjusted comparisons. Our negative findings for mineral intake differed from Nguyen and colleagues^9^ who found several minerals associated with high-risk depression for Japanese women but not men. They were sufficiently powered (*n* = 770) to find small effect size differences that may not be clinically meaningful. Their sample included women over 65 years old from one coastal suburb of Japan and they adjusted for age and other demographic variables as well as BMI and hypertension, but sleep and exercise were not considered. Other researchers examining minerals and depression have reported differences in zinc.^50–53^ The recommended intake for zinc is ≥8.0 mg/day^33^ and our sample averaged 7.0–7.5 mg/day with no significant group difference.

### Strengths and limitations

Our findings address a gap in knowledge about women in the decade prior to menopause, before acquiring additional risk of depression due to menopausal transition and hormonal fluctuations. The major strengths include participants in the same reproductive stage and rigorous dietary measures that involved detailed nutrition assessments with three dietary recalls surrounding an in-person visit for demographic and anthropometric measures rather than self-report. Our ethnically diverse sample was from California’s San Francisco Bay Area, with a temperate climate, access to fresh fruits and vegetables regardless of season, and a population generally considered healthy and educated, as reflected in our low smoking rates, number of college graduates, and level of physical activity. Given this somewhat unique sample, our results may not be generalizable to all premenopausal midlife women.

Like any secondary analysis, our dietary measures could have been more rigorous, particularly for types and amounts of fruits and vegetables. Nutrient intake was calculated from three diet recalls during a 1-week period and may not reflect a person’s typical diet. In addition to the small sample size, other limitations include absence of serum levels for nutrients, and our cross-sectional design not allowing for directionality.

A final limitation was the use of self-report dietary intake, particularly for alcohol consumption, where both our low-risk and high-risk groups would be considered light drinkers. While it may seem counter-intuitive that our high-risk depression group consumed significantly less alcohol, others report similar findings.^54^ Individuals with depressive symptoms may consciously or unconsciously limit alcohol intake because of its depressant effects. Alcohol intake may merely coincide with positive social interactions, as our group difference was no longer present after adjusting for demographic factors including a partner, thus supporting positive effects of socializing rather than any negative effect of alcohol.^54^

## Conclusion

In summary, 30% of our sample of women in midlife were at high risk for depression, even before onset of menopause when hormonal changes, weight gain, and social factors can further exacerbate symptoms. Interventions to address these symptoms prior to menopause may reduce or prevent major depression and need for pharmacological therapy. Dietary changes, such as limiting ultra-processed foods containing polyunsaturated fat, omega-6 fatty acids or sucrose, and encouraging omega-3 fatty acids and fiber may be one approach. Weight loss may be an additional strategy; however, it was lack of exercise and short sleep duration rather than BMI that consistently remained associated with high-risk depression in our participants. The mechanistic framework for depicting the interaction between nutrients and depression, proposed by Marx and colleagues,^24^ addresses obesity as either a cause or a consequence of depression. The complexity of these interactions make it difficult but critical to disentangle various mechanisms involved in depression so that appropriate therapeutics can be implemented. Our findings indicate that these factors likely interact on multiple levels, and interventions should be individually tailored, as one approach may be more acceptable than another. However, more research is needed to better understand the interactive roles of diet, exercise, and sleep in midlife women’s experience of depressive symptoms.

## Data Sharing Statement

Data described in the article, code book, and analytic code will be made available upon request to Dr. K.A.L., University of California San Francisco, San Francisco, CA, USA.

## Abbreviations Used

ALA: α-linolenic acid
BMI: Body mass index
CES-D: Center for Epidemiological Studies Depression Scale
DHA: Docosahexaenoic acid
EPA: Eicosapentaenoic acid
FSH: Follicle Stimulating Hormone
NCEP: National Cholesterol Educational Program
MEDFICTS: Represents the eight food categories of meat, eggs, dairy, fried foods, baked goods, convenience foods, table fats, and snacks
PPAQ: Paffenbarger Physical Activity Questionnaire
SD: Standard deviation
SPSS: Statistical Package for Social Sciences
SWAN: Study of Women Across the Nation
